# Crystallography and the development of therapeutic medicines

**DOI:** 10.1107/S2052252518002555

**Published:** 2018-02-26

**Authors:** Edward N. Baker

**Affiliations:** a School of Biological Sciences, University of Auckland, Private Bag 92-019, Auckland, New Zealand

**Keywords:** crystallography, therapeutic medicines, structure-guided drug development, editorial

## Abstract

Advances in crystallography and cryo-EM promise to further enhance the proud role crystallography has played in the development of new medicines.

One of the highlights of the 24th Congress of the International Union of Crystallography, held in Hyderabad (India) in August 2017, was the Ewald Prize lecture delivered by Professor Sir Tom Blundell. In it he stressed the social importance of crystallography, and its long history of use in the development of medicines, from the crystallization of insulin in the 1920s through to the widespread use of structure-guided drug development in the pharmaceutical industries and academia of today (Blundell, 2017 ▸).

During most of this time the focus has been on single-crystal studies of the appropriate biological targets, either at the earliest stages of discovery where fragment-based studies can be used to identify initial drug candidates (Erlanson *et al.*, 2016 ▸), or at later stages where drugs can be optimized through iterative rounds of structural analysis of protein–drug complexes. These approaches are eloquently described by Blundell (2017 ▸). Probably the most spectacular example of these approaches was the successful development, in the face of a virtual worldwide panic over the seemingly untreatable AIDS epidemic, of a large repertoire of anti-HIV drugs targeted against key HIV proteins: the HIV protease, reverse transcriptase and integrase enzymes. Crystallography, in both the pharmaceutical industry and academia, was central to this achievement, and if a Nobel Prize could be awarded to a scientific discipline, this would have been one for crystallography. Subsequent successes have included the development of anti-influenza drugs targeted against the viral neuraminidase enzyme, and many more are in the pipeline.

There are limitations in the application of single-crystal studies to drug discovery, however (Rawson *et al.*, 2017 ▸). The biological targets are generally individual proteins (particularly membrane proteins) or larger protein assemblies or protein/nucleic acid complexes such as the ribosome. Many may not be accessible to crystallography, either because they cannot be expressed in sufficient quantities, or because they cannot readily be crystallized (Carpenter *et al.*, 2008 ▸). The exciting recent advances in cryo-EM, which have brought revolutionary improvements in the resolution and clarity that can be attained for EM maps (Kühlbrandt, 2014 ▸; Subramaniam *et al.*, 2016 ▸), seem certain to change all this. There have already been a number of reports of cryo-EM structures in which bound ligands are quite well defined (*e.g.* Banerjee *et al.*, 2016 ▸; Gao *et al.*, 2016 ▸); their locations are clear as are their effects on the surrounding structure. How often high enough resolution can be attained to identify hydrogen bonds and bound water molecules – important aspects of drug optimization – is less certain. Time will tell.

The potential utility of cryo-EM in drug discovery and its relationship with crystallographic studies is addressed in an article in this issue of **IUCrJ**, in which Amporndanai *et al.* (2018 ▸) report a combined X-ray and cryo-EM investigation of a key membrane protein complex, cytochrome *bc*_1_, in complex with several lead compounds. Cytochrome *bc*_1_ is a validated drug target against the parasites *Plasmodium falciparum* and *Toxoplasma gondii*, the causative agents of malaria and toxoplasmosis, respectively. The native parasite proteins cannot be obtained in sufficient quantities for crystallography, however, and cytochrome *bc*_1_ proteins of other species must be used as a surrogate. Using bovine cytochrome *bc*_1_, the authors show that fairly similar resolutions can be attained from both the crystal structures and the EM maps, and the ligands are similarly bound, although neither gives atomic resolution.

While we cannot yet fully foresee how this will play out, the history of crystallography has been that as new structural methods become available (for example protein NMR in the 1980s) an equilibrium soon becomes established in which each approach adapts in ways that optimize their respective strengths. It is certain that cryo-EM will render many more important targets amenable to structure-informed drug discovery, especially for membrane proteins and large assemblies (Rawson *et al.*, 2017 ▸). Conventional crystallography, however, has great strengths in the resolution that can be attained and in its applicability to fragment screening and other high-throughput approaches. Ultimately it is sample quality and availability that will decide, but it seems to me that the two approaches will move forward together in a highly complementary way. A cryo-EM structure could be a very powerful guide to designing an optimal construct for crystallography, for example, when higher resolution is needed.

As Dimitri Argyriou has pointed out, crystallography in its broadest sense is constantly evolving (Argyriou, 2017 ▸). Not only does cryo-EM offer exciting new possibilities, but the development of X-ray free-electron lasers (XFELs), and of serial crystallography at both XFEL and advanced synchrotron sources, now allow us to ‘tackle with relative ease complex biological structures or to take crystallography to unthinkably small time-frames and nanocrystals’. Many of these advances have been reported in this journal, and suggest that these, too, will come to bear on drug discovery. Serial crystallography, for example, first developed at XFEL sources is now being applied at synchrotron facilities, where use of a mix-and-diffuse approach has shown promise for rapid, high-throughput, drug screening (Beyerlein *et al.*, 2017 ▸).

Finally, I return to the first application of crystallography in medicine, the use of crystallization as a quality control step in the preparation of insulin. A recent article points to the potential for using crystallization for quality control in the preparation of new biologics drugs (Brader *et al.*, 2017 ▸). These are primarily monoclonal antibodies such as the breast cancer drug Herceptin, and can be extremely effective. They are, however, very expensive because each new batch must be rigorously tested with a panel of biophysical tools. There is good reason to believe that crystallization and crystallography can be used to drastically lower these costs, giving yet another new application for crystallography.
